# Elucidation of the Cellular Interactome of African Swine Fever Virus Fusion Proteins and Identification of Potential Therapeutic Targets

**DOI:** 10.3390/v15051098

**Published:** 2023-04-29

**Authors:** Isabel García-Dorival, Miguel Ángel Cuesta-Geijo, Inmaculada Galindo, Ana del Puerto, Lucía Barrado-Gil, Jesús Urquiza, Covadonga Alonso

**Affiliations:** Departmento de Biotecnología, INIA-CSIC, Centro Nacional Instituto Nacional de Investigación y Tecnología Agraria y Alimentaria, Ctra. de la Coruña Km 7.5, 28040 Madrid, Spain; cuesta.miguelangel@inia.csic.es (M.Á.C.-G.); calonso@inia.csic.es (C.A.)

**Keywords:** virus–host interaction, interactome, drug target, lipid metabolism enzymes, fusion proteins, African swine fever virus, ASFV

## Abstract

African swine fever virus (ASFV) encodes more than 150 proteins, most of them of unknown function. We used a high-throughput proteomic analysis to elucidate the interactome of four ASFV proteins, which potentially mediate a critical step of the infection cycle, the fusion and endosomal exit of the virions. Using affinity purification and mass spectrometry, we were able to identify potential interacting partners for those ASFV proteins P34, E199L, MGF360-15R and E248R. Representative molecular pathways for these proteins were intracellular and Golgi vesicle transport, endoplasmic reticulum organization, lipid biosynthesis, and cholesterol metabolism. Rab geranyl geranylation emerged as a significant hit, and also Rab proteins, which are crucial regulators of the endocytic pathway and interactors of both p34 and E199L. Rab proteins co-ordinate a tight regulation of the endocytic pathway that is necessary for ASFV infection. Moreover, several interactors were proteins involved in the molecular exchange at ER membrane contacts. These ASFV fusion proteins shared interacting partners, suggesting potential common functions. Membrane trafficking and lipid metabolism were important categories, as we found significant interactions with several enzymes of the lipid metabolism. These targets were confirmed using specific inhibitors with antiviral effect in cell lines and macrophages.

## 1. Introduction

African swine fever (ASF) is a major threat against the swine industry due to its rapid distribution across Europe and Asia. Currently, ASF has spread worldwide since the first outbreak, and recently occurred in America in the Dominican Republic and Oceania, [1,2]. This lethal disease is caused by the African swine fever virus (ASFV), a large complex virus with a double-stranded DNA genome. It encodes for more than 150 ORFs, where the function of most of them is still unknown [3]. However, knowing the functions of the viral proteins and their potential cellular interactome is crucial for the rational development of new antiviral strategies.

There are several studies trying to characterize the relationship among several cellular proteins and ASFV proteins [4,5,6,7]. However, there are very few studies that investigate the interactome for those ASFV proteins [8,9].

Interestingly, there is some degree of homology of several ASFV proteins with the entry/fusion complex proteins of the closely related vaccinia virus (VACV) [5,10,11]. Therefore, ASFV fusion proteins might be predicted either by their location at the internal membrane of the virion and/or by their sequence/structural homology to VACV fusion proteins. This and other previous results from studies on ASFV biology [5,10,12] conducted us to select severalASFV proteins that might be involved in the entry/fusion step to study their interactome.

ASFV enters the cells by endocytosis and virions traffic the endocytic pathway from the plasma membrane to the inner cell at very early times of infection. Following this, internalization occurs from the plasma membrane to endocytic tubulo-vesicles, named early endosomes (EE), which harbor ASFV as early as 15 min after infection [12]. EE then mature into late endosomes (LE), which are capable of fusion with autophagosomes and lysosomes (LY). The fate of the degradative pathway is lysosomal degradation by hydrolases of the enclosed material. It is already known that small GTPases Rab5 and Rab7 are the master regulators of this traffic, being predominant at the limiting membrane of EE and LE/LY, respectively. Endosomal maturation entails the exchange of Rab proteins and other endosomal membrane molecules, and the acidification of the endosomal lumen. We have demonstrated that Rab7 is crucial for ASFV infection, and the LE is the relevant organelle for ASFV entry [12]. Moreover, our previous work demonstrated that, early upon infection, ASFV encounters the restriction of innate immune pathways also at the endocytic pathway [7].

Incoming ASF virions escape lysosomal degradation by egressing from the LE through a process called viral fusion. This is mediated by the interaction of two ASFV fusion proteins (E199L and E248R) with the Niemann-Pick C1 (NPC1) receptor [5] in order to reach the cytoplasm for replication [10]. The ASFV internal membrane fuses then with the LE limiting membrane in an ill-defined process that is crucial for most viruses trafficking the endosomal pathway. Then, viral fusion at endosomes would allow viral escape to the cytosol to start replication. We previously characterized how the ASFV initiates virion uncoating along its endosomal trafficking by taking advantage of some of the molecular features of these vesicles. In fact, ASFV capsid gets dissolved due to the acidification of endosomes [12] and exposes the viral internal membrane that could mediate fusion from then on. ASFV proteins pE248R and E199L, located at the virion internal membrane, are, apparently, some of the viral proteins responsible for mediating fusion during ASFV cycle [10].

Therefore, the study of the interactome of this virus and of some selected ASFV proteins will allow us not only to better understand the virus biology, but also to characterize and identify the biological functions of these viral proteins.

In this manuscript, we present a high-throughput proteomic analysis of four potential entry/fusion ASFV proteins to elucidate their interactome and potential function during the viral cycle by using affinity purification and label-free mass-spectrometry analysis. Furthermore, the common interactions of some viral proteins indicate that they could share functions, possibly important for the ASFV cycle and potential candidate targets for antivirals.

## 2. Materials and Methods

### 2.1. Construction of Plasmids That Express ASFV Proteins Fused to EGFP

Codon-optimized cDNA sequences encoding ASFV-BA71 proteins (NCBI reference sequence number: NC_001659) were cloned into the pEGFP-C1 plasmid to generate MGF360-15R, P34, E248R and E199L proteins with N-terminal EGFP tag; the plasmid sequences were confirmed by sequencing by GeneArt Technologies (Thermo Fisher Scientific; Waltham, MA, USA).

### 2.2. Cell Culture and Transfections

Vero cell line (ATCC CCL-81) and human embryonic kidney 293T (HEK 293T; ATCC CRL-3216) cells were grown in Dulbecco’s modified Eagle’s medium (DMEM) supplemented with 5 or 10% heat-inactivated fetal bovine serum (FBS), respectively, 1% glutamax and 1% penicillin–streptomycin (Gibco, Thermo Fisher Scientific; Waltham, MA, USA) at 37 °C with 5% CO_2_. Swine alveolar macrophages were collected by alveolar lavage with phosphate-buffered saline (PBS) as previously described [13] and cultured at 37 °C in RPMI medium containing inactivated 10% swine serum, 2 mM L-glutamine, 50 μM 2-mercaptoethanol, 20 mM Hepes and 30 μg/mL gentamycin. To transfect 293T cells, four 60 cm^2^ dishes were seeded with 3 × 10^6^ cells 24 h prior to lipofectamine transfection with 4 μg of plasmid DNA encoding the different ASFV-BA71 proteins and 9 μL of lipofectamine 2000 (Life Technologies, Carlsbad, CA, USA). After 24 h post-transfection, the cells were harvested, lysed and immunoprecipitated using a GFP-Trap system (Chromotek/Chromotek & Proteintech; Planegg-Martinsried, Germany) according to the manufacturing protocol.

### 2.3. EGFP Coimmunoprecipitations

Some of the methodology described in this paper was described by García-Dorival et al. [14,15]. EGFP-MGF360-15R, EGFP-P34, EGFP-E248R, EGFP-E199L and EGFP immunoprecipitations were performed using a GFP-Trap_A system (Chromotek/Chromotek & Proteintech; Planegg-Martinsried, Germany) according to the manufacturing protocol. The cell pellet was resuspended in 200 μL of lysis buffer (10 mM Tris/ Cl pH 7.5; 150 mM NaCl; 0.5 mM EDTA; 0.5%NP40) supplemented with Halt Protease Inhibitor Cocktail EDTA-Free (Thermo Fisher Scientific; Waltham, MA, USA) and incubated for 30 min on ice. The lysate was then clarified by centrifugation at 14,000× *g* and diluted five-fold with dilution buffer (10 mM Tris/Cl pH 7.5; 150 mM NaCl; 0.5 mM EDTA) supplemented with Halt Protease Inhibitor Cocktail EDTA-Free (Thermo Fisher Scientific; Waltham, MA, USA). The GFP-Trap agarose beads were equilibrated with ice-cold dilution buffer supplemented with Halt Protease Inhibitor Cocktail EDTA-Free and then incubated with diluted cell lysate overnight at 4 °C on a rotator, followed by centrifugation at 2500× *g* for 5 min. The bead pellet was washed two times with wash buffer (10 mM Tris/Cl pH 7.5; 150 mM NaCl; 0.5 mM EDTA) supplemented with Halt Protease Inhibitor Cocktail EDTA-Free. After the removal of wash buffer, the beads were resuspended with 100 μL of (2×) SDS Buffer (Invitrogen, Thermo Fisher Scientific; Waltham, MA, USA) and incubated for 10 min at 95 °C in a thermal shaker to elute bound proteins; the beads were then collected by centrifugation and the eluted proteins were transferred to a 1.5 mL centrifuge tube. Immunoprecipitated samples were then analyzed using label-free mass spectrometry.

### 2.4. Sample Digestion

In gel, digestion was essentially performed as described by Shevchenko et al., 2006 [16]. Samples (20 µL, in reducing sample buffer) were run approximately 1 cm into a 4–12% NuPage gel (Thermo Fisher Scientific; Waltham, MA, USA) before staining with Coomassie blue (GelCode Blue Safe Protein Stain, Fisher Scientific, Thermo Fisher Scientific; Waltham, MA, USA) for at least 1 h (h), then de-stained with ultrapure water for at least 2 h. The entire lane length (1 mm wide) was excised and cut into smaller pieces (approx. 1 mm^3^) before de-staining with 25 mM ammonium bicarbonate/50% acetonitrile (*v/v*). Proteins were reduced for 10 min at 60 °C with 10 mM dithiothreitol in 25 mM ammonium bicarbonate and then alkylated with 55 mM iodoacetamide (both from Sigma-Aldrich/Merck; Darmstadt, Germany) in 50 mM ammonium bicarbonate for 30 min in the dark at room temperature. Gel pieces were washed for 15 min in 50 mM ammonium bicarbonate and then dehydrated with 100% acetonitrile. Acetonitrile was removed and the gel plugs rehydrated with 0.01 µg/µL proteomic grade trypsin (Thermo Fisher Scientific; Waltham, MA, USA) in 25 mM ammonium bicarbonate. Digestion was performed overnight at 37 °C. Peptides were extracted with 50% (*v/v*) acetonitrile, 0.1% TFA (*v/v*) and the extracts were reduced to dryness using a centrifugal vacuum concentrator (Eppendorf, Hamburg, Germany) and resuspended in 3 % (*v/v*) methanol, 0.1 % (*v/v*) TFA for analysis by MS.

### 2.5. Mass Spectrometry

LC-MS/MS analysis was similar to that described before [17]. Peptides were analyzed by on-line nanoflow LC using the Ultimate 3000 nano system (Dionex/Thermo Fisher Scientific, Waltham, MA, USA). Samples were loaded onto a trap column (Acclaim PepMap 100, 2 cm × 75 μm inner diameter, C18, 3 μm, 100 Å) at 12 μL/min with an aqueous solution containing 0.1% (*v/v*) TFA and 2% (*v/v*) acetonitrile. After 7 min, the trap column was set in line with an analytical column (Easy-Spray PepMap^®^ RSLC 50 cm × 75 μm inner diameter, C18, 2 μm, 100 Å) fused to a silica nano-electrospray emitter also from Dionex. The column was operated at a constant temperature of 35 °C and the LC system coupled to a Q-Exactive HF mass spectrometer (Thermo Fisher Scientific; Waltham, MA, USA). Chromatography was performed with a buffer system consisting of 0.1% formic acid (buffer A) and 80% acetonitrile in 0.1% formic acid (buffer B). The peptides were separated by a linear gradient of 3.8–50% buffer B over 30 min at a flow rate of 300 nL/min. The Q-Exactive HF was operated in data-dependent mode with survey scans acquired at a resolution of 60,000 at m/z 200. Up to the top 16 most abundant isotope patterns with charge states +2 to +5 from the survey scan were selected with an isolation window of 2.0 Th and fragmented by higher energy collisional dissociation with normalized collision energies of 29. The maximum ion injection times for the survey scan and the MS/MS scans were 100 and 45 ms, respectively, and the ion target value was set to 3 × 10^6^ for survey scans and 1 × 10^5^ for the MS/MS scans. MS/MS events were acquired at a resolution of 30,000. Repetitive sequencing of peptides was minimized through dynamic exclusion of the sequenced peptides for 20 s.

### 2.6. Mass Spectrometry Data Analysis

MS spectra data were analyzed by label-free quantification using the MaxQuant software v 1.6.17.0 [18] and searched against a human protein database (Uniprot UP000005640_9606) and the ASFV bait protein sequence (Uniprot UP000000624) using the Andromeda search engine. The false discovery rate (FDR) was set to 0.01, and a decoy database was included in the search to help identify and remove false positives. MQ results were further processed with the online tools PERSEUS (version 2.0.6.0) from Max Plank Institute [19] to highlight significant interacting proteins. PERSEUS analysis was conducted as described by Garcia-Dorival et al., 2014 and 2016.

### 2.7. Identification of the Interacting Partners of ASFV Proteins

Label-free mass spectrometry results were processed and analyzed using the Perseus software (MaxQuant); this software was used to perform the statistical analysis and to differentiate background proteins (those cellular proteins that interacted with EGFP alone) from interacting proteins (those cellular proteins that interacted with EGFP-MGF360-15R, EGFP-P34, EGFP-E248R or EGFP-E199L). LFQ intensity values were analyzed using Perseus software platform, version 2.0.6.0 [19], as described before [14,15]. Proteins identified by a single peptide were removed and *p* value threshold was set at <0.05 for the *t*-test analysis. From this analysis, a volcano plot graphic and a table showing the interacting partners were generated for each ASFV viral protein showing the statistically significant proteins, those proteins that had a high probability of interacting with each viral protein.

### 2.8. Identification of Protein Networks and Common and Unique Pathways in the Interactomes Using Software Analysis

To generate interpretable outputs for the different interactome datasets, we used Metascape software, a gene annotation and analysis resource for the interpretation and understanding of large datasets. It provided automated meta-analysis tools to understand either common or unique pathways and protein networks as well [20].

Metascape is an efficient tool to comprehensively analyze and interpret OMICs-based studies in the big data [20]. We used Express Analysis, which takes a streamlined approach by running the analysis steps with popular default settings [20].

For each given gene list, pathway and process enrichment analysis has been carried out with the following ontology sources: KEGG Pathway, GO Biological Processes, Reactome Gene Sets, Canonical Pathways, Cell Type Signatures, CORUM, TRRUST, DisGeNET, PaGenBase, Transcription Factor Targets, WikiPathways, PANTHER Pathway and COVID [20]. All genes in the genome have been used as the enrichment background. Terms with a *p*-value < 0.01, a minimum count of 3 and an enrichment factor >1.5 (the enrichment factor is the ratio between the observed counts and the counts expected by chance) were collected and grouped into clusters based on their membership similarities. More specifically, *p*-values were calculated based on the cumulative hypergeometric distribution [21] and Q-values were calculated using the Benjamini–Hochberg procedure to account for multiple testings [22]. Kappa scores [23] were used as the similarity metric when performing hierarchical clustering on the enriched terms, and sub-trees with a similarity of >0.3 were considered a cluster. The most statistically significant term within a cluster was chosen to represent the cluster [20].

For a functional analysis of each viral interactome, each list of the protein interacting partners was first uploaded into the Metascape program. Then, we performed a pathway and process enrichment analysis. To do this, we first identified statistically enriched terms (including GO/KEGG terms, canonical pathways, hallmark gene sets, etc., based on the default choices under Express Analysis), accumulative hypergeometric *p*-values and enrichment factors were calculated and used for filtering. Remaining significant terms were then hierarchically clustered into a tree based on Kappa-statistical similarities among their gene memberships (such as NCI DAVID site). Then, 0.3 Kappa score was applied as the threshold to cast the tree into term clusters [20].

In addition, a protein–protein interaction enrichment analysis was performed. To do this, for each given interactome list, protein–protein interaction (PPI) enrichment analysis was carried out with the following databases: STRING, BioGrid, OmniPath and InWeb_IM. Only physical interactions in STRING and BioGrid were used. The resultant network contains the subset of proteins that form physical interactions with at least one other member in the list [20]. 

### 2.9. Validation of the MS Results

To validate the MS results, a transfection of the plasmids following the immune precipitation method was performed. Beads were then resuspended and boiled in 100 μL of 2× SDS sample buffer at 95 °C for 10 min to elute the bound proteins. This was similar to what was carried out for the MS sample preparation. The beads were then collected by centrifugation and SDS- PAGE was performed with the supernatant (elution faction). To perform the SDS-PAGE, 10% SDS polyacrylamide gels were used; then, gels were transferred to PVDF membranes (Millipore/Merck; Darmstadt, Germany) using a semidry transfer system. Transferred membranes were blocked in 10% skimmed milk powder dissolved in TBS-0.1% Tween (TBS-T) (50 mM Tris-HCl (pH 8.3), 150 mM NaCl and 0.5% (*v/v*) Tween-20) buffer for 60 min at room temperature. Primary antibody was diluted 1:1000 in blocking buffer and then incubated at 4 °C overnight. The rabbit antibodies used were anti-Rab5 (1:1000) anti-Rab7 (1:1000) anti-Rab 11 (1:2000) from cell signaling, anti-NPC1 (1:1000, Abcam, Abcam, Cambridge, United Kingdom), anti-SREBP2 (1:1000, Novus Biologicals, Zillow, CO, USA), anti-SACM1L (1:1000, Proteintech, Rosemont, IL, USA), anti-VAP-A and anti-VAP-B (1:1000), anti-ABCD3 (1:1000). The latter three antibodies from Atlas antibodies Bromma, Sweden, anti-ORP1L (1:1000, Novus Biologicals, Zillow, CO, USA) and anti-PI4Kß (Merck, Darmstadt, Germany). The mouse antibodies used were anti-GFP (1:1000), anti-DDX3 (1:1000), the latter two antibodies from Santa Cruz Biotechnology, Dallas, TX, USA, anti-Tubulin (1:2000, Sigma-Aldrich/Merck; Darmstadt, Germany), anti-GAPDH (1:5000, Abcam, Cambridge, United Kingdom) and anti-p30 antibody, used at 1:1000. After three washes, blots were incubated with appropriate HRP secondary antibody diluted in blocking buffer at a 1:5000 and incubated for 1 h at room temperature. Finally, blots were then developed using enhanced chemiluminescence reagent (BioRad, Hercules, CA, USA) and detected using a BioRad Imaging system.

### 2.10. Viruses and Infection

We used the cell culture adapted and nonpathogenic ASFV isolate Ba71V that is adapted to grow in the Vero cell line [24]. We obtained the recombinant virus (ASFV B54GFP) from parental Ba71V, which expresses GFP as a p54 fusion protein [25]. ASFV viral stocks were propagated and titrated by plaque assay in Vero cells, as previously described [24]. For immunofluorescence, ASFV stocks were partially purified using a 40% sucrose cushion in PBS at 68,000× *g* for 50 min at 4 °C and were further used at an MOI of 1 unless otherwise indicated.

### 2.11. Indirect Immunofluorescence and Antibodies, Conventional and Confocal Microscopy

Cells were seeded onto 12 mm glass coverslips prior to ASFV infection. Cells were then washed with PBS and fixed with 4% formaldehyde or 4% paraformaldehyde for 15 min. After washing with PBS, the cells were then incubated with 50 mM NH4Cl in PBS for 10 min. Then, coverslips were incubated in blocking buffer (0.1% saponin, 0.5% BSA in PBS) for 1 h. Coverslips were then incubated for 1 h in specific primary antibodies diluted in blocking buffer at 37 °C. The following rabbit antibodies were used: anti-SREBP2 (Novus Biologicals, Zillow, CO, USA), anti-SACM1L (Proteintech, Rosemont, IL, USA) and anti-PI4Kß (Merck, Darmstadt, Germany), all antibodies used at 1:50 concentration for immunofluorescence. Mouse monoclonal antibodies were: p72 (1BC11) and p150 (17AH2), both from Ingenasa, Gold Standad Diagnosis; Madrid, Spain) used at 1:100 and CD63 (Novus biologicals) at 1:50 concentration. The appropriate secondary antibody conjugated to Alexa Fluor −488 or −594 were used and cell nuclei detected with TOPRO3.Coverslips were mounted on glass slides using ProLong Gold. The conjugated secondary antibodies and the latter two were from Thermo Fisher Scientific; (Waltham, MA, USA). Cells were imaged using a TCS SPE confocal microscope (Leica, Wetzlar, Germany) with a 63× oil immersion objective. Image acquisition was performed with a Leica Application Suite Advanced Fluorescence Software (LAS AF). All the images were acquired at a resolution of 1024 × 1024 pixels.

Image analyses were performed with Leica Application Suite advanced fluorescence software (LAS AF) and ImageJ software.

### 2.12. Experiments with Inhibitors

Antiviral compounds used included Atorvastatin (ATV; 300 μM and 100 μM), a HMG-CoA reductase inhibitor [26], and Ezetimibe (EZT; 50 μM and 25 μM), an NPC1 inhibitor of clinical use as a cholesterol-lowering drug [27], Cilostazol (CLZ; 500 μM and 250 μM), an unrelated enzyme inhibitor, and a phosphodiesterase 3 inhibitor [28,29,30]. Moreover, we used specific inhibitors: an inhibitor of enzyme carnitine palmitoyltransferase (CPT; ST1326; 50 μM and 100 μM; [31]), named Triacsin C (TC; 25 μM and 50 μM), and an inhibitor of the activity of the enzymes Acyl-CoA Synthetase Long (ACSL) Chain Family Member 3 and 4 [32].

Vero cells and macrophages were pretreated with inhibitors at the indicated concentrations, followed by infection with B54GFP at a moi of 1 pfu/cell. Recombinant B54GFP is a recombinant ASFV expressing green fluorescent protein (GFP) as a fusion protein of viral p54 [25]. Cells were washed with PBS after 60 min of adsorption at 37 °C and incubated 16 h with DMEM 2%. Then, cells were harvested by trypsinization and washed with flow cytometry buffer (PBS, 0.01% sodium azide and 0.1% bovine serum albumin). Detection of ASFV-infected cells was performed in an FACS Canto II flow cytometer (BD Sciences). To determine the percentage of infected cells per condition, 10,000 cells/time point were scored and analyzed. Infected cell percentages obtained after drug treatments were normalized to control values.

All compounds used were purchased from Sigma. Previously, we analyzed cell viability and performed cytotoxicity tests with all inhibitors using the CellTiter 96 Non-radioactive Cell Proliferation Assay (Promega, Madison, WI, USA) following the manufacturer’s instructions. We also studied the cytotoxic activity of the organic solvent DMSO. Based on these experiments, we selected optimal nontoxic working concentrations for infection assays.

## 3. Results

### 3.1. Expression of ASFV VP in Cells

To determine the cellular interacting partners of the four ASFV viral proteins (VP) analyzed in this study, we used quantitative proteomics coupled to an immunoprecipitation strategy. This strategy was based on expressing ASFV VP as EGFP fusion proteins in 293T cells and using a high-affinity GFP coimmunoprecipitation system (GFP-Trap; Chromotek, /Chromotek & Proteintech; Planegg-Martinsried, Germany). While the target proteins were expressed as EGFP fusion proteins, the expression of EGFP alone was used as a control. This technique has been shown to improve the discrimination between specific and nonspecific interactions to the target proteins [15,33,34].

**Figure 1:**
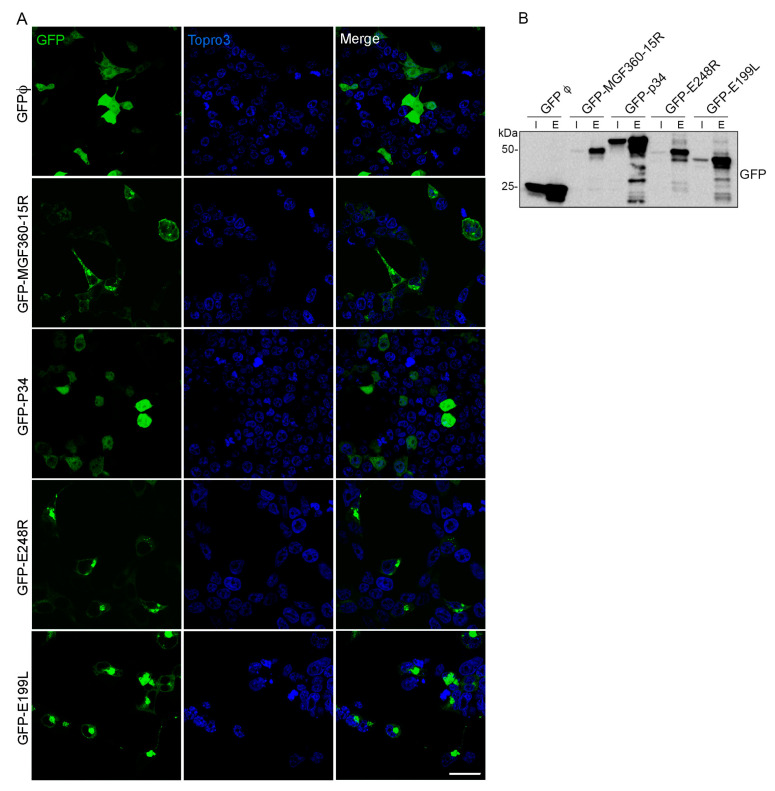
Expression and coimmunoprecipitation of GFP-tagged ASFV proteins (GFP-VP). (**A**) Expression of ASFV proteins MGF360-15R, P34, E248R, E199L and control GFP in HEK 293T cells as confirmed by immunofluorescence. As expected, cells showed expression of GFP (upper panel) and diverse distribution of viral proteins GFP-MGF360-15R, GFP-P34, GFP-A278R and GFP-E199L. GFP, Topro3 and Merge images are indicated in upper panels in colors. Scale bar 25 µm. (**B**) WB detection of GFP-tagged ASFV viral proteins MGF360-15R, P34, E248R, E199L and control GFP in the immunoprecipitation assay. “I” refers to the input sample and “E” to the elution sample. Cell lysates from transfected cells were co-immunoprecipitated with agarose beads.

Once overexpressed in cells (Figure 1A), fusion proteins were extracted from lysed cells and coimmunoprecipitated using the GFP-Trap system. These cells derived from embryonic kidney cells were selected due to their high transfection efficiencies, which enhances statistical significance of the assay. Another important reason to select this cell line is its complete annotated genome in databases, which is crucial for protein identification and function assignment. Both input (cell lysate) and eluted fractions (from the coimmunoprecipitation) were analyzed by Western blot (Figure 1B). The data indicated that both proteins P34 and E199L were expressed with high efficiency, while ASFV proteins MGF 360-15R and E248R were not expressed at similarly high levels. Expressed proteins were detected at the expected molecular weights, namely EGFP-P34~63 kDa, EGFP-E199L~49 kDa, EGFP-MGF360-15R~59 kDa and EGFP-E248R~55 kDa.

### 3.2. Identification of the Potential Cellular Interacting Partners of MGF360-15R, P34, E248R and E199L ASFV Proteins

To identify interacting cellular partners specific for ASFV proteins MGF360-15R, P34, E248R and E199L, we used label-free LC−MS/MS [14,15,35,36].

Once ASFV viral proteins MGF360-15R, P34, E248R and E199L fused to EGFP and control EGFP were overexpressed in 293T cells and their cellular binding partners immunoprecipitated using the GFP-trap system, the immunoprecipitated proteins were identified by mass spectrometry. To differentiate the nonspecific interactions, a statistical analysis was performed in triplicate [14,15].

**Figure 2:**
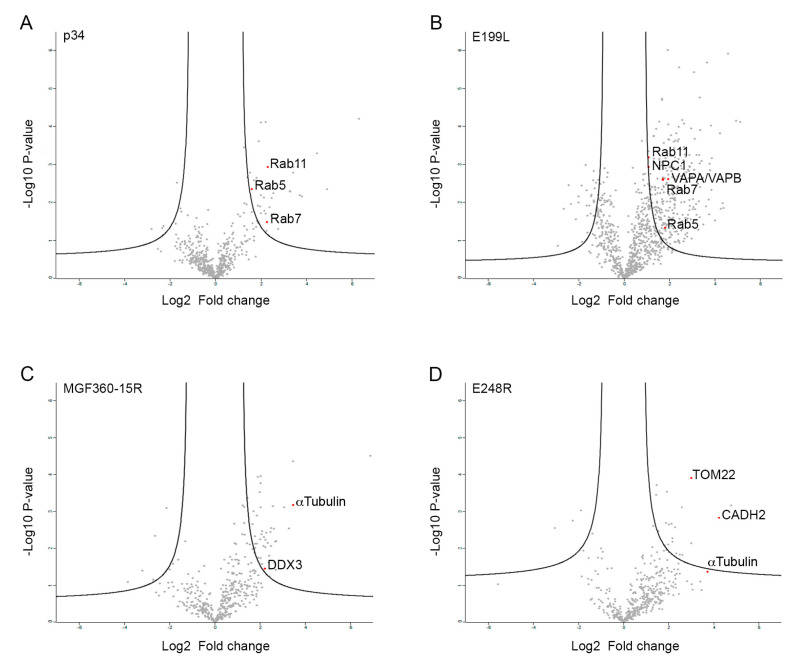
Volcano plots representing mass spectrometry and statistical analysis results for ASFV-tagged viral proteins. Analysis by MS included EGFP-tagged ASFV proteins and control. The immunoprecipitation and label–free mass spectrometry analysis was performed in triplicate. The logarithmic fold change is plotted against the negative logarithmic *p* values of the t test. In these volcano plots, the dashed curve indicates the region of significant interactions; the dots in the upper right quadrant represent potential protein-interacting partners for the following ASFV proteins: (**A**) EGFP-P34, (**B**) EGFP-E199L, (**C**) EGFP- MGF360-15R and (**D**) EGFP-E248R. For any potential protein interaction partner, the values of their abundance when coimmunoprecipitated with those EGFP–ASFV fusion proteins were compared to their value from the coimmunoprecipitation with the control (EGFP control). Dots corresponding to relevant hits from statistical analysis are highlighted in red.

The MS label-free datasets generated by Max Quant were then analyzed by the Perseus software (version 2.0.6.0), as described in the methodology section. From this analysis, a volcano plot graphic for EGFP-MGF360-15R, EGFP-P34, EGFP-E248R and EGFP-E199L (Figure 2A–D, respectively) was generated to show the proteins that had a high probability of interacting with each viral protein. Statistically significant proteins, with a fold change greater than 2, were found in the right quadrant.

For each ASFV viral protein analyzed in this study, between 30 and 200 binding partners, respectively, were identified as potential cellular interactors (Tables S1A, S1B, S1C and S1D). Some of the viral proteins shared common interactor cellular proteins. In the interactomes of the ASFV proteins P34 and E199L, we found approximately 20 interactome partners that these two viral proteins shared. Moreover, proteins MGF360-15R and E248R shared seven interactome partners. This could suggest that these proteins act co-ordinately upon infection.

### 3.3. Functional Analysis of the ASFV Interactome Proteins

**Figure 3:**
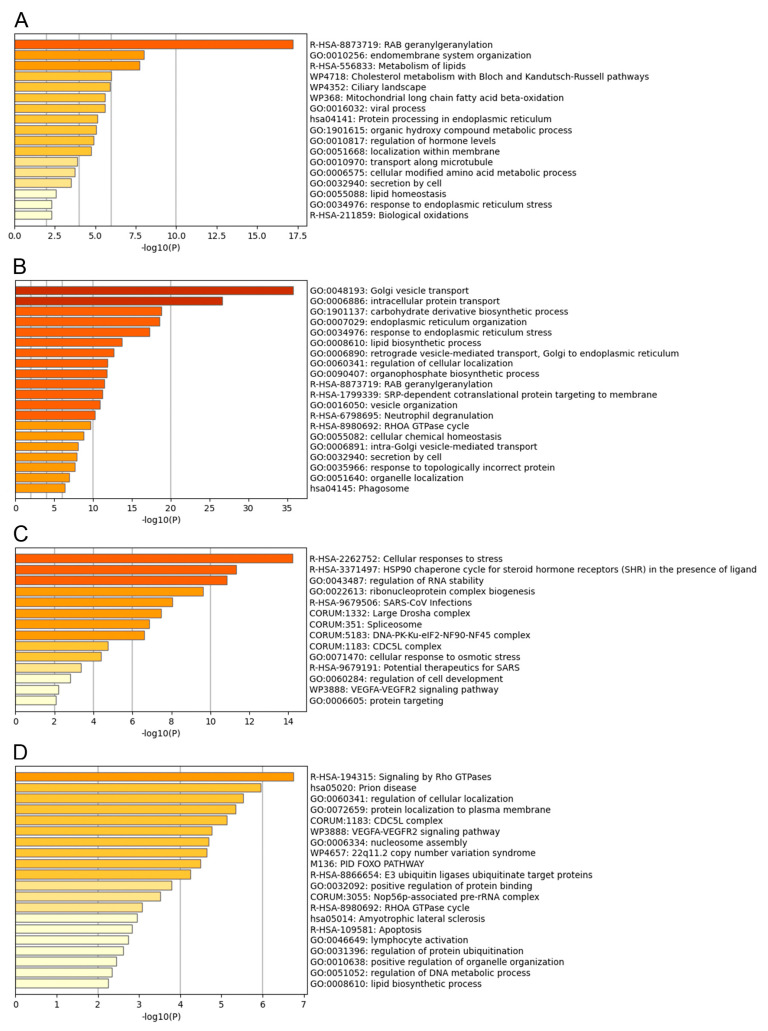
Bioinformatic and functional analysis of the ASFV interactome of proteins P34, E199L, MGF360-15R and E248R. Bar graph of enriched terms across input gene list, colored with intensities according to their *p*-values. Bar graph represents the top 20 enriched terms (such as GO/KEGG terms, canonical pathways, hall mark gene sets, etc.) across the different ASFV proteins, colored according to their –log10 (*p*-value) for proteins: (**A**) P34, (**B**) E199L, (**C**) MGF360-15R and (**D**) E248R. A complete list of the terms in each cluster can be found in the supporting information (Table S1).

**Figure 4:**
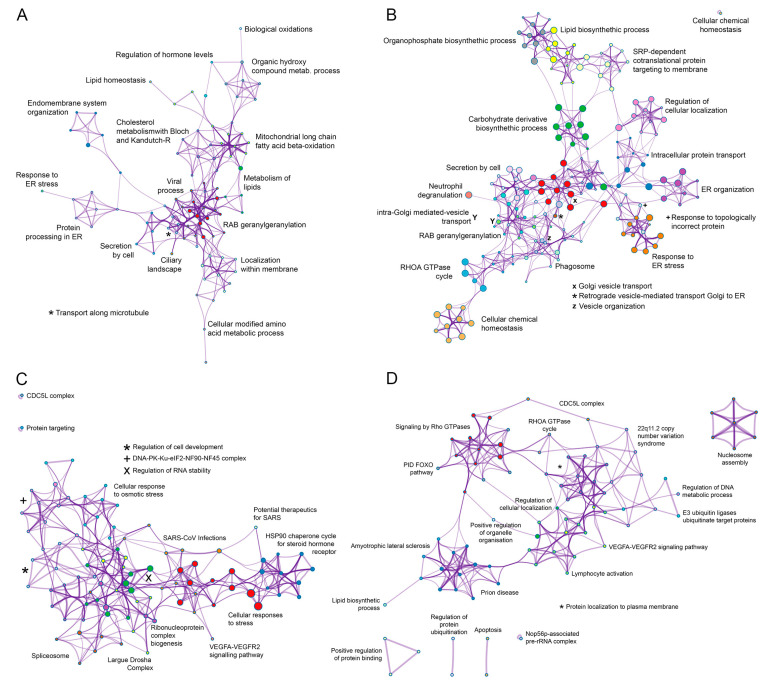
Network of enriched terms colored by cluster ID of the interactome for ASFV proteins (**A**) P34, (**B**) E199L, (**C**) MGF360-15R and (**D**) E248R. To further capture the relationships between the terms, a subset of representative terms from the full cluster was selected and converted as a network layout. More specifically, each term is represented by a circle node, where its size is proportional to the number of input genes falling under that term, and its color represents its cluster identity (i.e., nodes of the same color belong to the same cluster). For clarity, labels are only shown for one term per cluster.

Metascape program was used to identify pathways and to predict the protein−protein interactions of the statistically significant proteins and to group proteins in clusters according to function [20]. The top 20 clusters with their representative enriched terms of the P34, E199L, MGF360-15R and E248R ASFV viral proteins are displayed in Figure 3A–D, respectively. The single components for each enriched term are shown in Tables S2A to S2D. Furthermore, a network of the enriched terms colored by cluster ID of the interactome for ASFV proteins is also shown in Figure 4A–D, respectively.

For interactomes with a higher number of interacting partners, we were able to identify up to 100 enriched ontology clusters. This is the case of E199L viral protein, which yielded more than 200 cellular interactors and approximately 80 enriched ontology clusters (Figure S1).

Furthermore, for each interactome dataset, the protein–protein interactions (PPI) among input genes were extracted to form a PPI network. MCODE algorithm was then applied to identify neighborhoods where proteins were densely connected (Figure S2A–D). Finally, for each interactome database, GO enrichment analysis was applied to extract “biological meanings” (Figure S3A–D), where the top three best *p*-value terms were retained (Table S3D).

The most statistically enriched term for p34 was Rab geranyl geranylation, followed by metabolism of lipids and cholesterol, endoplasmic reticulum (ER) organization, protein processing, etc. (Figure 3A). The small GTPases of the Rab family are key regulators of intracellular membrane trafficking, being responsible for vesicle formation, movement and their fusion with membranes. Beyond that, they play a critical role in regulating exocytic and endocytic pathways. We found significant hits in P34 interactome, as shown in Table S1A, as the following: Rab11B, Rab5A, Rab5C, Rab2A, Rab4A, Rab7A, Rab21, Rab14, cholesterol metabolism-related scavenger Receptor Class B Member 1, SAC1-like phosphatidylinositide phosphatase (SACM1L), ADP ribosylation-factor-like (Arl) GTPase 6 and 8 interacting proteins, sphingolipid biosynthesis regulator ORMDL1, and the enzymes acyl-CoA synthetase long (ACSL) chain 3 and 4, and carnitine palmitoyltransferase 2 (CPT2). These enzymes were selected as potential drug targets.

Top statistically enriched terms for pE199L were Golgi vesicle transport, intracellular protein transport, carbohydrate and lipid biosynthetic process, ER organization and ER stress, Rab geranyl geranylation, etc. (Figure 3B), followed by a total of 80 enriched ontology clusters with 200 cellular interactors (Figure S1). Several of these pathways have been related to the establishment of the niche of replication of several viruses [37], as will be discussed below. ERGIC1, ER membrane proteins, TMEM33, Derlin, scavenger receptor class B member 2, mannose-6-phosphate receptor, Rab GTPases 7, 6, 3, 2, 14, 5, 1, VAPB and A, or SACM1L were significant hits of pE199L, to mention some examples (Table S1B).

Cellular responses to stress, chaperones for steroid receptors, several categories related to RNA, spliceosome and cdc5L complex were the top significant terms that clustered cellular interactors for MGF360-15R (Figure 3C). Categories related to cellular response to stress are common cellular responses to viral infections. Tubulin and LARP1 were also found in this protein interactome (Table S1C).

E248R interactors clustered under the statistically significant terms related to cdc5L complex, VEGFA-R2 and Rho GTPases signaling pathways (Figure 3D). ASFV protein E248R interactome profile revealed several cellular cytoskeleton-related proteins, such as cadherin, actin, and tubulin, as well as, mitochondrial proteins ATP5F1C and TOMM22, reticulons, Lamin B receptor, derlin, afadin and chaperonin CCT4 that assist the folding of actin and tubulin. Importantly, in the E248R, we found TMEM33 shared with E199L and PI4K, the cellular phosphatidylinositol (PI) 4-kinase alpha (PI4K), a known host factor necessary for the replication of several viruses [38,39].

### 3.4. Common Interactomes and Pathways among ASFV Viral Proteins

**Figure 5:**
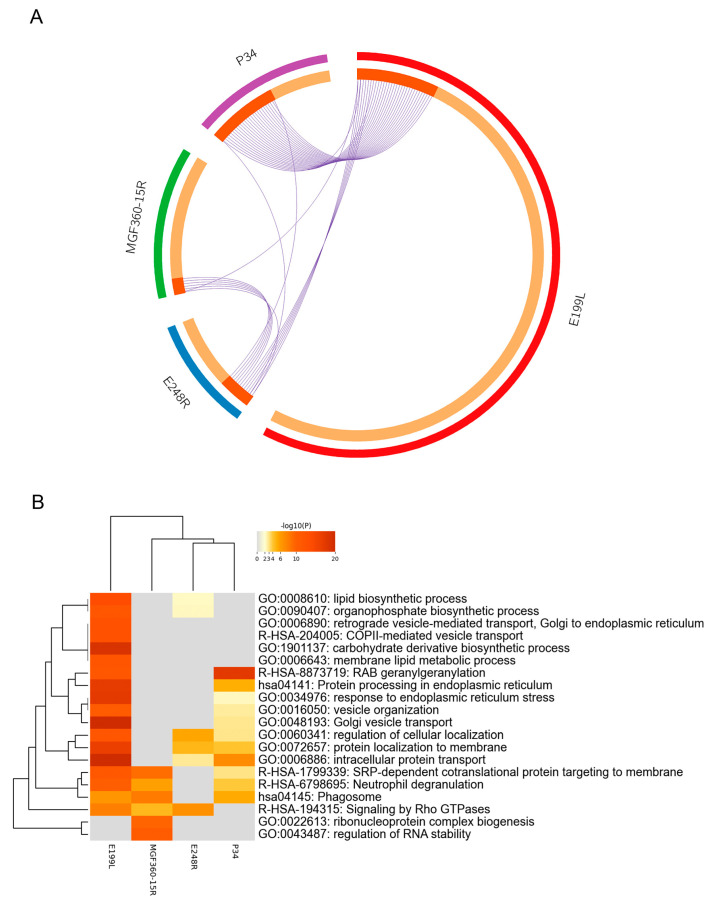
Visualizations of meta-analysis results based on multiple interactomes of the ASFV viral proteins (MGF360-15R, P34, E248R and E199L). (**A**) Circos plot shows the overlap of genes from the input gene list. On the inside, each arc represents one gene list. Dark orange color represents the genes that are shared by multiple lists and light orange color represents genes that are unique to a gene list. Purple lines link the same gene when shared by multiple gene lists. (**B**) Heatmap of enriched terms across the several input gene lists (MGF360-15R, P34, E248R and E199L) colored by *p*-values. The heatmap cells are colored by their *p*-values, and grey cells indicate the lack of enrichment for that term in the corresponding gene list.

After the analysis of each individual interactome dataset, another analysis was conducted comparing all four datasets to find potential common functions among viral proteins. To do this, a gene overlap analysis was first performed; the overlaps between these lists are shown in a Circos plot (Figure 5A). In this Circos plot, each viral protein was represented by a circle node, where its size is proportional to the number of input genes that fall under that specific viral protein.

**Figure 6:**
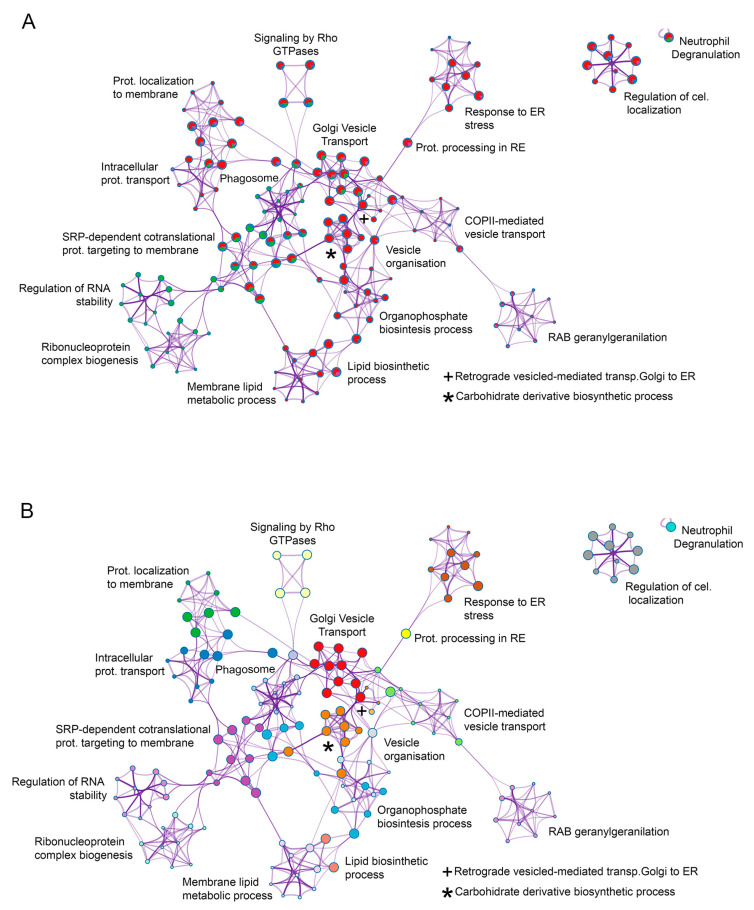
Network of enriched terms of meta-analysis results based on multiple interactomes of the ASFV viral proteins (MGF360-15R, P34, E248R and E199L). (**A**). Network of enriched terms represented as pie charts, where pies are color-based on the identities of the gene lists. Each pie sector is proportional to the number of hits originated from a gene list. (**B**). Network of enriched terms colored by cluster ID. To further capture the relationships between all the terms from the different gene lists, a subset of representative terms from the full cluster was selected and converted in a network layout.

A pathway process and enrichment analysis when comparing the four interactome datasets was conducted as well. In this case, to further capture the relationships between the terms, a subset of enriched terms was selected and rendered as a network plot. When multiple gene lists were provided, nodes were represented as pie charts, in which the size of a pie is proportional to the total number of hits that fall into that specific term. The pie charts are color-coded based on the gene list identities, where the size of a slice represents the percentage of genes under the term that originated from the corresponding gene list [20]. This plot is particularly useful for visualizing whether the terms are shared by multiple lists or they are unique to a specific list, as well as for understanding how these terms associate with each other within the biological context of the meta study (Figure 5B). These results are also shown as network meta-analysis results based on multiple interactomes of the ASFV viral proteins (MGF360-15R, P34, E248R and E199L (Figure 6A,B)).

As a result of this analysis, common interactomes among viral proteins were found. Such was the case of P34 and E199L, as these two viral proteins shared approximately 20 proteins, including Rab family GTPases Rab5, Rab7 and Rab11. Similar results have been found for viral proteins MGF360-15R and E248R, which shared approximately seven common cellular interactors, including Tubulin (Tub; Figure 2C,D).

### 3.5. Validation of MGF360-15R, P34, E248R and E199L Interaction Partners through Immunoprecipitation Analysis and Western Blot

**Figure 7:**
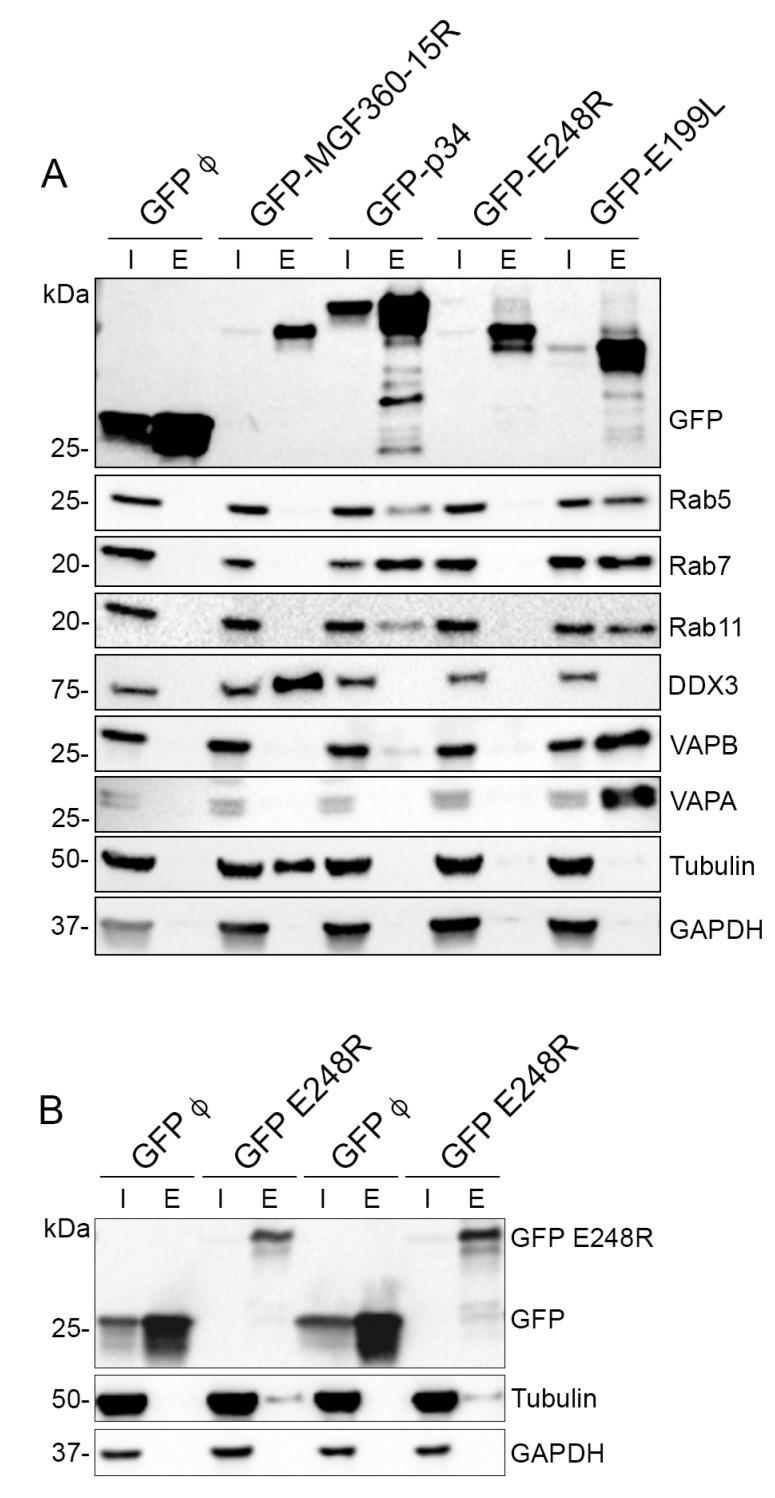
Immunoprecipitated GFP-tagged ASFV proteins with cellular proteins. Immunoprecipitated GFP-tagged ASFV viral proteins MGF360-15R, P34, E248R, E199L (EGFP-VP) and control EGFP, with the corresponding cellular proteins in the immunoprecipitation and MS assay. (**A**) WB detection of GFP-tagged ASFV viral proteins EGFP-MGF360-15R, EGFP-P34, EGFP-E248R and EGFP-E199L, control EGFP and selected cellular proteins that were identified by MS analysis, in the immunoprecipitation assay. “I” refers to the input sample and “E” to the elution sample. Immunoprecipitated cellular proteins were endogenous Rab5, Rab7, Rab11, DDX3, VAPB, VAPA, Tubulin alpha and GAPDH. Proteins were found at the expected molecular weights (MW). (**B**) As E248R had lower expression, we loaded a larger volume of sample per well (20 µL), and we could observe that this protein immunoprecipitated cellular tubulin alpha.

Selected cellular targets previously identified as interacting partners of ASFV viral proteins MGF360-15R, P34, E248R and E199L were then investigated using Western blot (WB) analysis on repeated immunoprecipitations using GFP-Trap as an alternative means of identification (Figure 7A,B). The selected cellular targets investigated for the ASFV interactome were Rab5, Rab7, Rab11, DDX3, VAPA, VAPB and Tubulin. These targets were selected due to their fold change value and because some of them were common interactors among the different viral proteins studied. Such was the case of Rab proteins found as common interactors for E199L and p34 (Figure 7A), or Tubulin, a common interactor for A276R and E248R (Figure 7A,B). To do this, the whole cell lysates (input) and immunoprecipitated samples (elution) from the immunoprecipitation analysis were analyzed by WB using a primary antibody against each selected protein as described above. The cellular protein GAPDH was used as a negative control as well as a loading control (Figure 7A).

**Figure 8:**
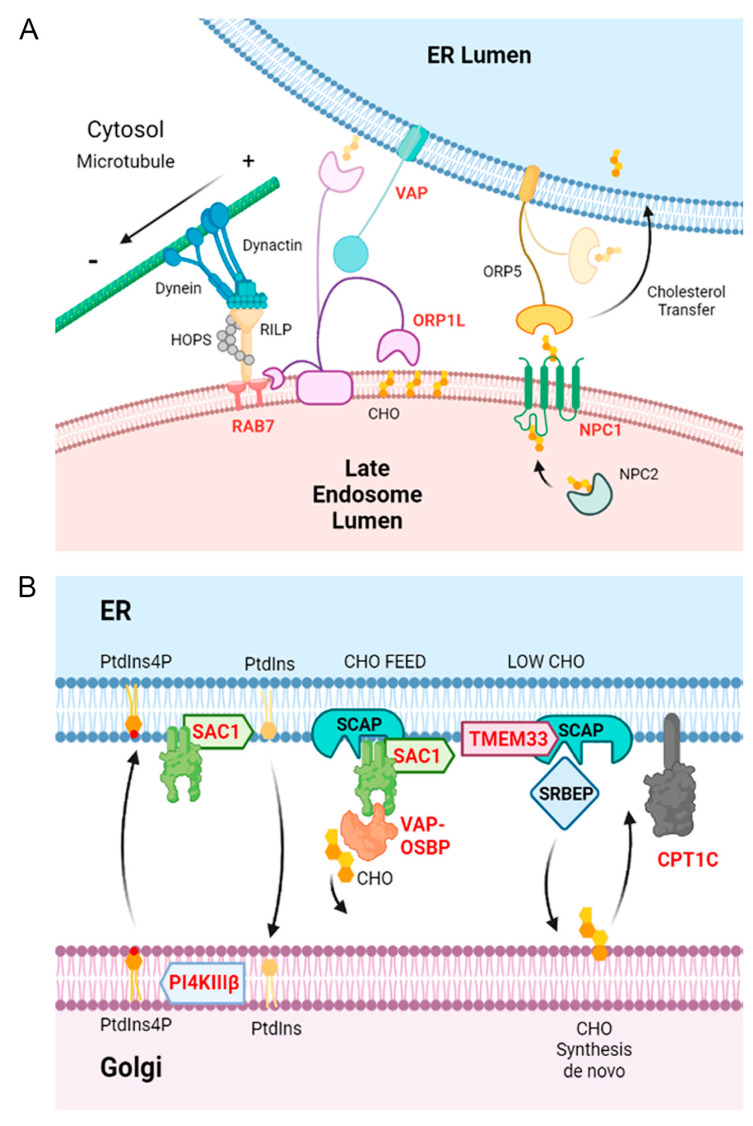
ASFV interactome significant hits related to Golgi transport and ER pathways. ER membrane contacts are important in the control of membrane trafficking and regulation of intracellular organelles. ASFV interactome significant hits are highlighted in bold letters in the schematics. (**A**). Upon stress and high cholesterol content, endosomes and lysosomes move to a perinuclear location clustered around the microtubule-organizing center together with vesicles of the trans-Golgi network (TGN). This movement is orchestrated by ER VAP that regulates the association (or dissociation) of ORP1L, in complex with RILP, Rab7, and ORP1L and the HOPS complex through microtubule motor dynein, as shown in panel (**A**). (**B**). Lipid transfer proteins regulate cholesterol transfer between ER and Golgi membranes, mediated by SACM1, OSBP and VAP. SACM1 is an ER-resident phosphatase that dephosphorylates PtdIns4P in the ER. It is also regulated by the carnitine palmitoyltransferase (CPT).

This immunoprecipitation analysis evidenced the presence of the interacting cellular proteins Rab5 in the elution samples of p34 and E199L (Figure 7A). This is the characteristic marker of early endosomes. Moreover, we could demonstrate immunoprecipitation of Rab7, the characteristic marker of late endosomes, and both proteins immunoprecipitated Rab11, a characteristic marker of recycling endosomes. This could indicate the relevance of these ASFV proteins for the endosomal traffic of the virus. Finally, p34 and pE199L also immunoprecipitated VAPB. Moreover, we were also able to demonstrate immunoprecipitation of the helicase DDX3 for MGF360-15R and the cytoskeletal protein tubulin for pE248R and MGF360-15R (Figure 7B). The vesicle-associated membrane protein-associated protein (VAP) family consists of five transmembrane ER proteins present on the membranes of various organelles to form membrane contact sites or MCSs [40]; VAP proteins are responsible of sterol transfer at MCSs, as will be explained below (Figure 8).

**Figure 9:**
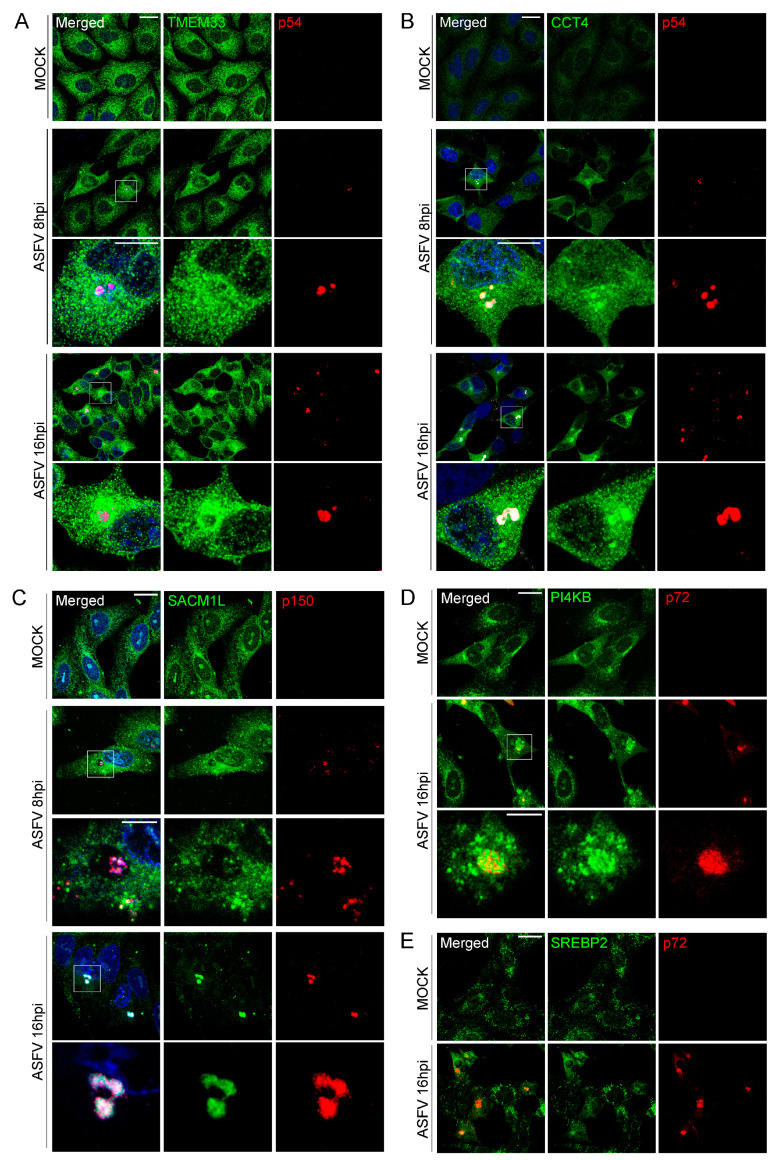
Lipid traffic-related proteins and ASFV infection. Immunofluorescence confocal images of mock (upper panel) or ASFV-infected cells at 8 (intermediate panel) and 16 hpi (lower panels). Lipid traffic-related proteins SACM1L, PI4K, SREBP and TMEM33 localized at or around ASFV replication sites (VF) as stained with a monoclonal antibody against ASFV p150 protein and rabbit antibodies against: (**A**) TMEM33, (**B**) CCT4, (**C**) SACM1L, (**D**) PI4K and (**E**). SREBP2, respectively, at the time points indicated. Scale bar: 20 µm. Zoom: 5 µm.

Moreover, using a confocal microscopy analysis of those proteins (Figure 9), we found a preferential localization for some of these hits at or around the viral factories from early (8 hpi) to late (16 hpi) times after infection. This was the case of the endoplasmic reticulum protein TMEM33, the chaperonin CCT4, PI4Kβ, the phosphoinositide converting enzyme PI4Kβ, which was a significant hit found in the pE248R interactome, and the SACM1L phosphatase, both regulating the PtdIns4P between organelle membranes and related protein SRBEP2. These proteins were reorganized to the viral replication sites at 8 hpi (SACM1L) and 16 hpi, respectively (PI4K, SREBP2). Moreover, we analyzed by Western blot the expression of these and other proteins related to lipid transport at the ER–Golgi membrane contacts (Figure S4), and we could observe a significant decrease in SRBEP2 expression at 16 hpi (*p* < 0.001) in triplicates. Jointly, the reorganization to the viral factories of these intracellular proteins responsible for membrane contact and transfer of SACM1L, PI4K and SRBEP2 could indicate their relevance for the formation of the viral replication sites.

### 3.6. Inhibition of Lipid Metabolism and Other Significant Pathways in Vero Cells and Macrophages

**Figure 10:**
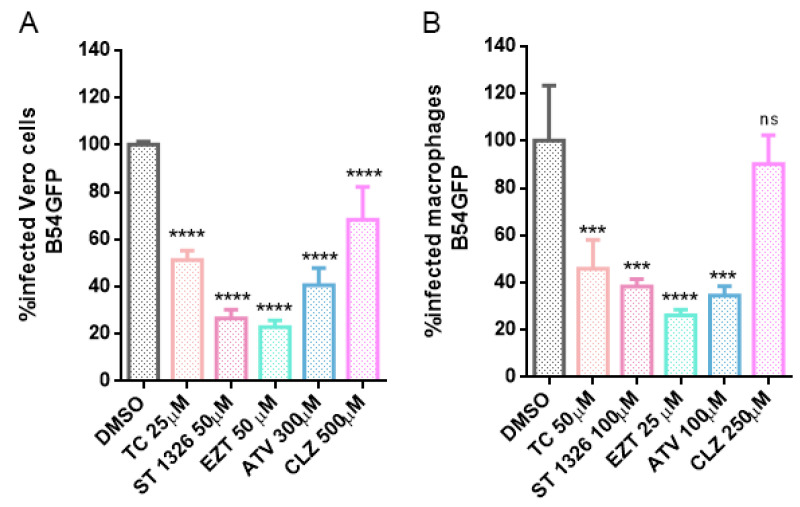
ASFV infection and inhibitors of lipid metabolism in Vero cells and macrophages. Inhibition of infection efficiency in cells treated with compounds inhibiting lipid metabolism. Inhibitor or DMSO pretreated ASFV-infected Vero cells (**A**) or macrophages (**B**) for 16 hpi, as detected by flow cytometry of cells infected with B54GFP and depicted as median and standard deviations in histograms. Statistically significant data are shown with asterisks (**** *p* < 0.0001, *** *p* < 0.001 and ns non-significant). The following lipid inhibitor compounds were used: ATV, atorvastatin; EZT ezetimibe; CLZ, cilostazol, ST1326; and TC, triacsin-C.

To further validate functionally the mass spectrometry results and the potential interacting partners of ASFV viral proteins MGF360-15R, P34, E248R and E199L, small molecule inhibitors were selected against some of the selected cellular proteins to test their effect on the ASFV viral life cycle in Vero cell line (Figure 10A) and in primary macrophages (Figure 10B). To evaluate ASFV infectivity by flow cytometry, we used a fluorescent recombinant ASF virus, BP54GFP, in which GFP is expressed late at infection as a fusion protein of p54.

Lipid biosynthesis and membrane lipid metabolic process were significant categories for E199L. Several enzymes important for the lipid metabolism were found as significant hits for p34 too. Lipid metabolism was an important category found in our interactome results; then, we selected inhibitor drugs targeting its related enzymes that could eventually inhibit ASFV (Figure 10A,B). We used statins, which are HMG-CoA reductase inhibitors, such as atorvastatin (ATV; 300 μM and 100 μM), as a generic lipid metabolism inhibitor, ezetimibe (EZT; 50 μM and 25 μM), an NPC1 inhibitor of clinical use, and cilostazol, a phosphodiesterase 3 inhibitor as an unrelated lipid metabolism enzyme, (CLZ; 500 μM and 250 μM). Moreover, we found as potential interactors of P34 the lipid metabolism enzymes carnitine palmitoyltransferase (CPT) and the Acyl-CoA Synthetase Long (ACSL) Chain Family Member 3 and 4. These enzymes are required for both synthesis and degradation of cellular lipids via beta-oxidation. Once the noncytotoxic working concentration was selected, we tested the effect of all these compounds on infectivity in Vero cells and macrophages compared with DMSO treatment using fluorescent recombinant virus B54GFP and flow cytometry. The specific inhibitor of CPT enzyme (ST1326; 50 μM and 100 μM) significantly inhibited ASFV infectivity, corroborating its relevance in ASFV infection (Figure 10A,B). Similarly, the inhibitor Triacsin-C inhibited infectivity of recombinant virus B54GFP (TC; 25 μM and 50 μM). Moreover, generic inhibitors, such as atorvastatin and ezetimibe, inhibited ASFV infectivity significantly at the doses used (**** *p* < 0.0001), but inhibitors of other enzymes, such as cilostazol, were not able to reduce infection over 15% or the data were not significant. This specific effect found with lipid metabolism inhibitors in macrophages ultimately corroborated the selectivity of the interactome found in this work.

## 4. Discussion

The interactome of the selected potential ASFV entry/fusion proteins illustrates how ASFV hijacks molecular pathways required for its trafficking at infection. Some of these results confirm previous findings [12,41] and altogether provide novel information to identify new potential therapeutic targets to develop antivirals.

We have studied the interactome of four of the ASFV proteins that were found to be homologous to the VACV fusion complex, namely, viral proteins, MGF360-15R, p34, E199L and E248R [10,42,43]. For the fusion protein E199L, we have found approximately 200 potential cellular interacting partners, which correspond to the categories, endosomal maturation and homeostasis, intracellular transport, Golgi vesicle transport, vesicle and endoplasmic reticulum organization, lipid biosynthesis, and membrane lipid metabolic process, among others. Some of these cellular hits and pathways have been already identified as relevant on the ASFV cycle in previous studies, such as Rab7 and phosphoinositides-related proteins, which were highlighted several years ago [12]. Surprisingly, some of these hits were also shared by other ASFV putative fusion protein, P34. Similarities were also found when comparing the interactomes of another pair of ASFV proteins, MGF360-15R and E248R. Several hits were shared between both viral proteins and this constitutes the first description of ASFV proteins sharing common cellular interactomes. Furthermore, this also highlights the importance of some pathways during the ASFV cycle, as more than one viral protein could target the same cellular hit. This information is crucial to select potential candidates for drug development against this disease.

According to our results, p34 and E199L interactomes were organized under the following most significant categories: Rab geranyl geranylation, ER membrane organization, metabolism of lipids, CHO metabolism and Golgi vesicle transport between others. It has been described that small GTPases of the Rab family are key regulators of intracellular membrane trafficking, co-ordinators of vesicle formation, maturation, movement and fusion with membranes, being critical regulators of exocytic and endocytic pathways [44,45,46], thereby corroborating the importance of these pathways for ASFV [12].

In addition to this, the similarities between the interactome of ASFV proteins in pairs might indicate that they could act co-ordinately to bind endocytic pathway regulators, thereby ensuring a tight regulation of the endocytic pathway. Moreover, the interaction of both E199L and P34 with Rab 5, Rab 7 and Rab 11 found by AP-MS was corroborated by immunoprecipitation. The selective function of these proteins provided some hints regarding their possible function at ASFV infection. For example, Rab 5 recruits to the membrane of early endosomes, different sets of downstream effectors directly responsible of vesicle formation, movement, tethering and fusion, such as EEA1 [47]. In fact, the ASFV virion colocalizes with EEA1 at early post-infection times [12] and, at later time points, colocalization of the viral cores with Rab 7 positive late endosomes has been reported.

In addition to this, trafficking these compartments appears to be critical for ASFV cell entry as dominant negative Rab7 mutant reduced the infection drastically [12]. Rab7 regulates the traffic of late endosomes and their fusion with lysosomes. Moreover, it governs early-to-late endosomal maturation, microtubule minus-end as well as plus-end directed endosomal migration and positioning, lipid metabolism and endosome–lysosome transport through different protein–protein interaction cascades [44,48]. Taken together, the integrity of these functions seems to be critical early at ASFV infection.

Physiological interactors of Rab 7 are RILP [49], OSBP [50] and the Rab escort protein 1 or CHM choroideremia protein [51], which is the substrate-binding subunit of the Rab geranylgeranyltransferase complex. This is another molecule regulating this pathway found in the p34 interactome, which is necessary for Rab proteins prenylation. Once the prenylation reaction is completed, their lipid anchors ensure that Rabs would be directed to the relevant cell membrane [52].

Another interacting partner for E199L, the small Rab GTPase Rab 11, participates in endocytic recycling, regulates constitutive and regulated secretion and apical recycling of several transmembrane proteins, and is required for melanosome transport and release. Additionally, this GTPase also regulates vacuolar ATPase intracellular transport, which is necessary for endosomal acidification [53], a prerequisite for ASFV infection [12]. For its functions, Rab 11 interacts PI4K [54], which was also found as an interacting partner for ASFV E248R in this study.

In addition, we have found a significant number of ER-related proteins as potential cellular interactomes of the ASFV fusion proteins analyzed in this study. Some of them were molecular elements present at the ER membrane contact sites, known to regulate ER contacts with other organelles, especially with endosomes. In fact, we found that several hits were related to the regulation of endosome positioning. The formation of ER contacts depends on the ER proteins VAPA and B. VAP stands for vesicle-associated membrane protein-associated proteins (VAP) A and B [55]. Their function is explained below and in Figure 8A schematics. These molecules are critical for the physiological transfer at endosome–ER contact sites.

Additionally, the endosome positioning process entails a constant balance between motor-directed transport along microtubules [44,56,57], and it is also dependent on cholesterol levels [58]. At high cholesterol levels, the dynein-binding endosomal protein RILP forms a complex with Rab7 and the cholesterol-binding protein ORP1L, promoting anterograde transport to the perinuclear area. Under low endosomal cholesterol concentration, endosomes are tethered to the ER by ORP1L binding to VAPA, leading to the dissociation of dynein from RILP [59]. Both VAPA/B are implicated with OSBP and SACM1L in the transfer of phosphoinositides at the endosome–ER membrane contacts [60]. Thus, ER constitutes a platform for the organization of the required motor proteins co-ordinated by the ER-resident protein VAPA and, ultimately, of endosomal positioning.

Importantly, we found that both viral proteins p34 and E199L were interactors of VAP proteins. In fact, subtype VAPB interacted with high significance with p34 and E199L, while VAPA was found to interact with E199L. The importance of VAP proteins has been described for other viruses, such as hepatitis C virus (HCV), in which the nonstructural protein NS5A and B have been shown to interact with VAPB [61]. It was reported that HCV proteins colocalize with VAPB at the ER and Golgi apparatus to facilitate viral replication as well. In this study, we found a similar distribution and colocalization in ASFV-infected cells. Apart from being implicated in endosome transport, VAPA and B also participate in the ER unfolded protein response (UPR), the calcium homeostasis regulation and the traffic between ER and Golgi at the membrane contact sites [62].

Lipid metabolism was also found as an important category for ASFV in the E199L and P34 interactome analysis. For p34, we found a high-significance interaction with the Acyl-CoA Synthetase Long (ACSL) Chain Family Member 3 and 4, an enzyme required for both synthesis of cellular lipids and degradation via beta-oxidation [63,64]. In fact, a specific inhibitor of this enzyme is Triacsin-C, which caused an inhibition of ASFV infectivity, which ultimately indicates the essential function of this protein for ASFV infection [32]. Moreover, it is known that statins, lipid-lowering drugs acting as HMG-CoA reductase inhibitors, inhibit some viral infections [26]. In fact, lovastatin [65] and atorvastatin (in this manuscript) were both able to inhibit ASFV infection.

Another cellular interacting partner for p34 was the enzyme carnitine palmitoyltransferase CPT2, which directly interacts with CPT1 [66], an ER-resident pseudo enzyme (Figure 8B). Under nutrient-rich conditions, it promotes anterograde endosome transport and blocks it under cellular stress conditions [67]. Hence, it promotes endosomal transport depending on nutrient availability in concert with proteins regulating lipid metabolism. The functional relevance of this enzyme in ASFV infection was confirmed, given that the specific CPT inhibitor ST1326 [31,68] strongly impaired infection efficiency, expanding the possibilities of potential antivirals against this virus. This enzyme acts as a regulator of SACM1L phosphatase activity. SACM1L mediates lipid transfer between closely opposed ER and endosomal membranes with several other lipid transfer proteins [69]. Under basal conditions, CPT1C inhibits SACM1L activity to maintain normal levels of PtdIns4P in the Golgi, allowing nutrient receptor trafficking [70].

Another significant interacting partner for E199L was TMEM33, which is an ER-stress-inducible protein that regulates ER structure by binding reticulons and Arl6lP1, which ultimately facilitates calcium flux from the ER to the cytosol [71,72]. Reticulons were frequently found as interactors of ASFV fusion proteins as shown here. TMEM33 acts as another cholesterol (CHO) sensor at the ER membrane depending on low and high CHO levels, as is depicted in Figure 8B. Furthermore, TMEM33 interacts with SCAP, which ultimately interacts with SACM1L [62]. At low ER CHO content, TMEM33 interacts with SCAP that escorts SREBP to the Golgi and cleaves and releases active SREBP for nuclear transcription, resulting in CHO synthesis de novo [73]. This appears as a relevant metabolic pathway in ASFV infection. Our time course experiments of ASFV infection showed a significant decrease in the expression of cellular SREBP at 16 hpi by Western blot compared with control cell levels (*** *p* < 0.001). Moreover, SACM1L distributed characteristically to the ER and Golgi apparatus in control cells but concentrated at the viral factories in infected cells.

We have also shown that E199L interacts with the intracellular cholesterol transporter Niemann–Pick-type C1 (NPC1) by using immunoprecipitation techniques [5]. These results further validate this analysis and gave confidence to our work. Additionally, the NPC1 inhibitor ezetimibe had a functional impact on ASFV infection, causing reductions around 70%, as shown here. In contrast, inhibitors of other related molecules, such as cilostazol, a phosphodiesterase 3 inhibitor, did not have remarkable impact on infection.

In previous studies, we have found that antivirals targeting several endosomal receptors are able to inhibit not only ASFV but other highly pathogenic virus infections [74]. We have found a relevant interaction of E199L with the endosomal protein NPC1 [5] that was confirmed in this study while elucidating the E199L interactome. Importantly, several publications reported that NPC1 was also a relevant protein for other viruses, such as EBOV [75,76], SARS-CoV-2 [77] and HIV [78], among others. This highlights the importance of the study of the viral interactome to elucidate new potential therapeutic targets and common interactomes among different viruses.

Taken together, the interactome of these ASFV fusion proteins, highlighted potential functions on the regulation of ASFV endosomal traffic, positioning, fusion and exit. Some of the most significant hits were coincident with molecules required for building the viral replication site of other viruses (e.g., hepatitis C) and the redirection of vesicular traffic towards the viral factory. The most significant hits were part of signaling pathways related to cellular transport, the ER–Golgi transport and the regulation of several arms of the lipid transport and metabolism. In fact, drugs targeting specific enzymes of lipid metabolism found as hits were active in reducing ASFV infectivity in cells, highlighting the relevance of ASFV control of lipid metabolism. Several of the significant hits were molecular elements of the ER–membrane contacts and ER stress signaling, among others. Overall, this study illustrates the multifunctionality of viral proteins and offers a wealth of useful targets to develop new and more specific antivirals.

## Data Availability

All data are contained in the manuscript, Figures and Supplementary Figures.

## Supplementary Materials

The following supporting information can be downloaded at https://www.mdpi.com/article/10.3390/v15051098/s1. Figure S1: Bioinformatic and functional analysis of the ASFV interactome of E199L protein. Bar graph of the top 100 enriched terms across input gene list, colored with intensities according to their *p*-values. Bar graph represents the top 100 enriched terms (such as GO/KEGG terms, canonical pathways, hall mark gene sets, etc.) for E199L ASFV protein, colored according to their –log10 (*p*-value); Figure S2: Protein-protein interaction (PPI) network identified in the interactome lists for (A) P34, (B) E199L, (C) MGF360-15R, and (D) E248R. All protein-protein interactions among input lists were extracted from PPI data source and formed a PPI network. GO enrichment analysis was applied to the network to extract “biological meanings”. For each interactome list protein-protein interaction enrichment analysis was carried out with the following databases: STRING, BioGrid, OmniPath, InWeb_IM, using only physical interactions in STRING and BioGrid. The resultant network contains the subset of proteins that form physical interactions with at least one other member in the list; Figure S3: MCODE components identified for the interactome for (A) E199L, (B) P34, (C) MGF360-15R, and (D) E248R. After the protein-protein interaction (PPI) enrichment analysis, the Molecular Complex Detection (MCODE) algorithm was then applied to this network to identify neighborhoods where proteins are densely connected. If the network contains between 3 and 500 proteins, MCODE algorithm has been applied to identify densely connected network components. The MCODE networks identified for individual gene lists have been gathered and are shown in for each ASFV protein. Figure S4: Lipid traffic-related proteins at membrane contact sites were detected at sequential post infection times (1, 3, 6 and 16 hpi), using antibodies against A. SREBP2, SACM1L, ACBD3, PI4Kß, ORP1L, and ASFV p30 in triplicates, and B. CDH2, DDX3, CCT4 and p72 in duplicates. Statistically significant differences, such as a reduction of expression, was found for SREBP2 at 16 hpi. Tubulin and GAPDH were used as load controls. Figure S5: Membranes used to compose Figure 4. A. Membranes for Figure 4A were revealed with mouse antibodies anti-GFP, anti-alpha tubulin, anti-GAPDH, and anti-DDX3, and rabbit antibodies anti-Rab5, anti-Rab7, anti-Rab11, anti-SACM1L, anti-VAPA, and anti-VAPB. B. Membranes for Figure 4B were revealed with mouse antibodies anti-GFP, anti-alpha tubulin, and anti-GAPDH antibodies. Figure S6: Membranes used to compose Figure S4A. Membranes were revealed with mouse antibodies anti-p30, anti-GAPDH, and rabbit antibodies anti-SREBP2, anti-ACBD3, anti-PI4KB, anti-SACM1L, and anti-ORP1L. Figure S7: Membranes used to compose Figure S4B. Membranes were revealed with mouse antibodies anti-p30, anti-p72, anti-DDX3, anti-GAPDH, anti-CH2, anti-CT4 and anti-TMEM33 antibodies.
