# Supplementary figures and images for: Elucidation of the Cellular Interactome of African Swine Fever Virus Fusion Proteins and Identification of Potential Therapeutic Targets
Source: Viruses. 2023 Apr 29;15(5):1098. doi: 10.3390/v15051098 (PMC10221787; doi:10.3390/v15051098)

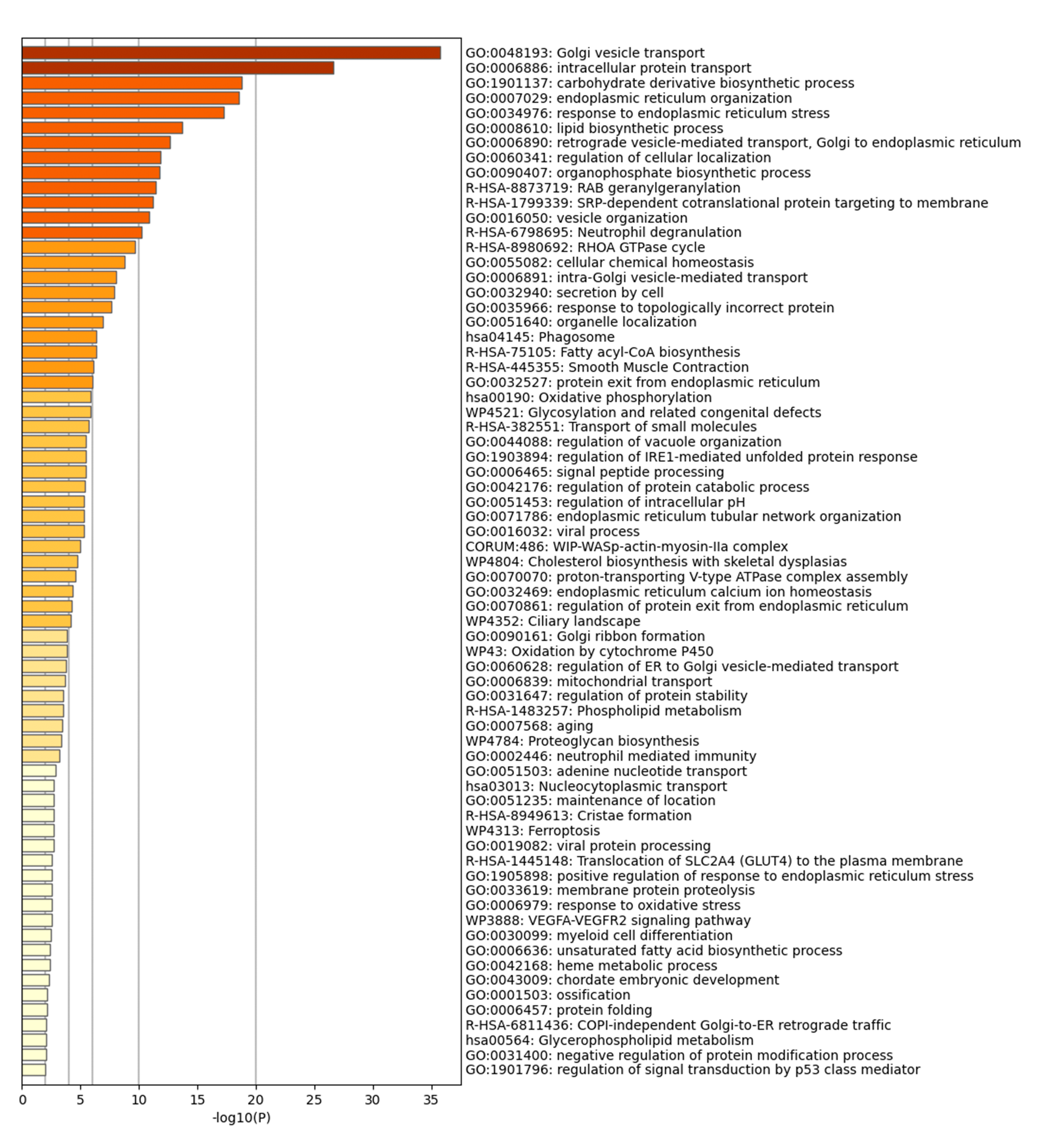

Supplement: Supplementary file 1 [file viruses-15-01098-s001.zip › Figure S1.tif]

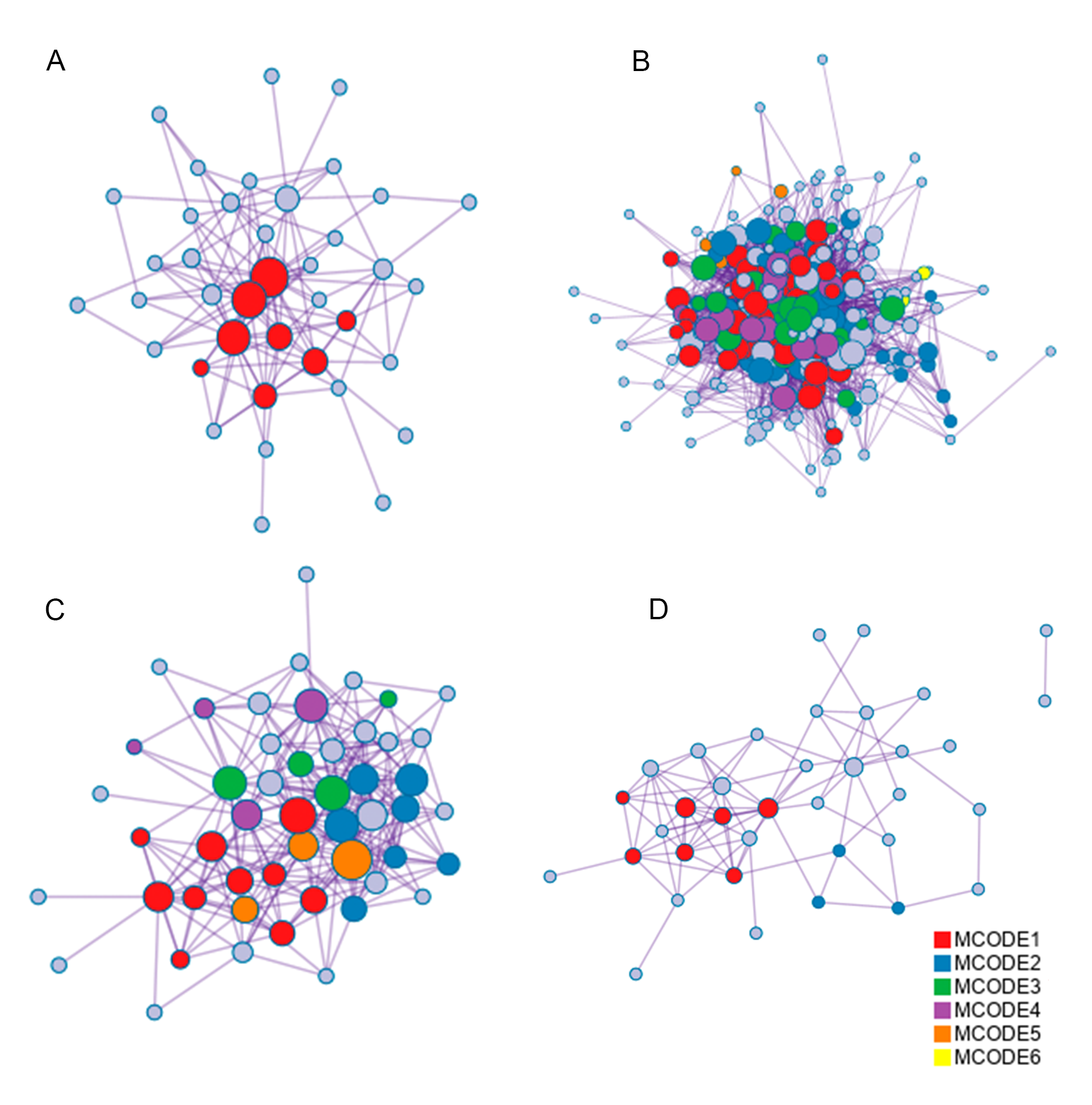

Supplement: Supplementary file 1 [file viruses-15-01098-s001.zip › Figure S2.tif]

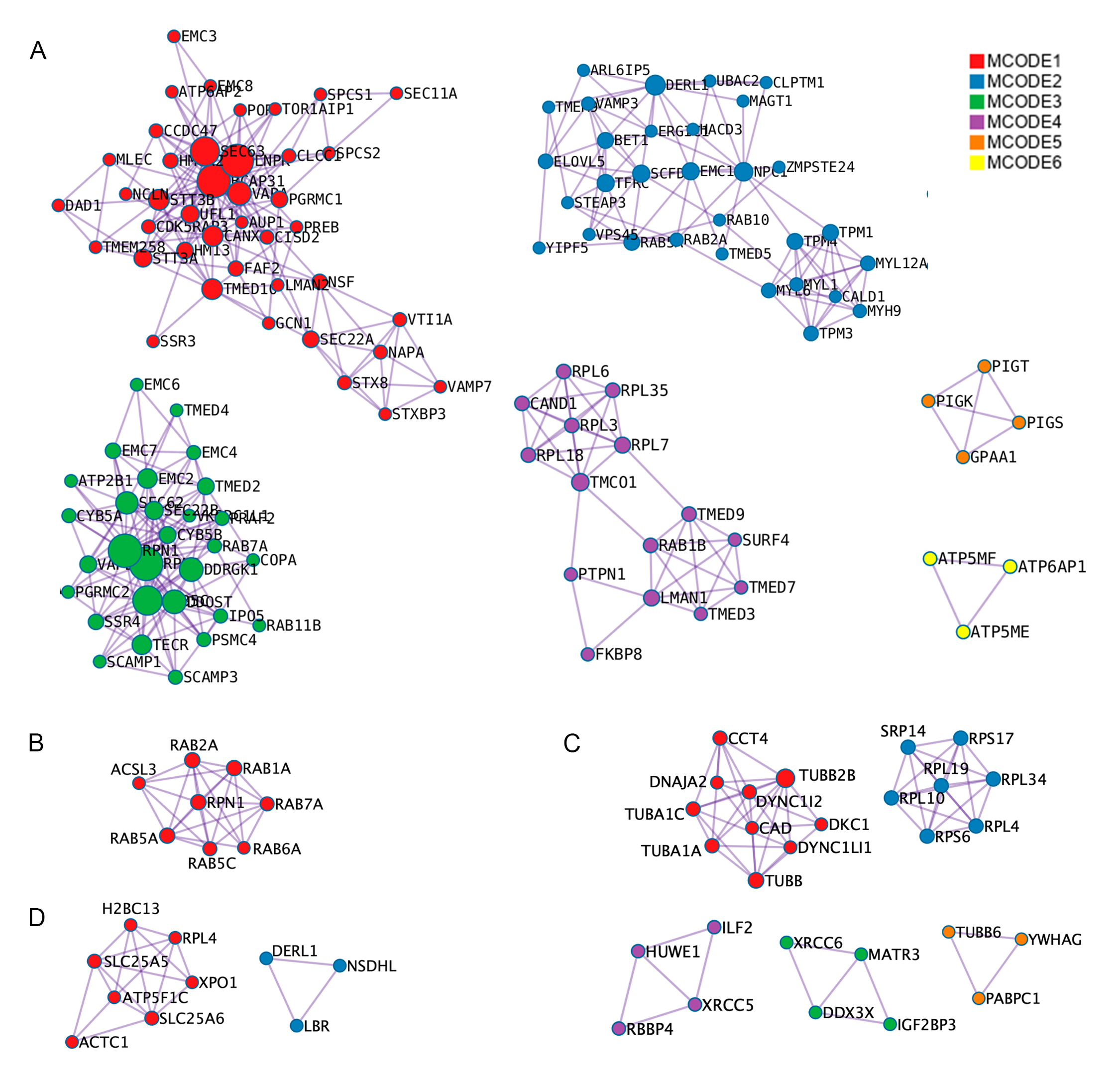

Supplement: Supplementary file 1 [file viruses-15-01098-s001.zip › Figure S3.tif]

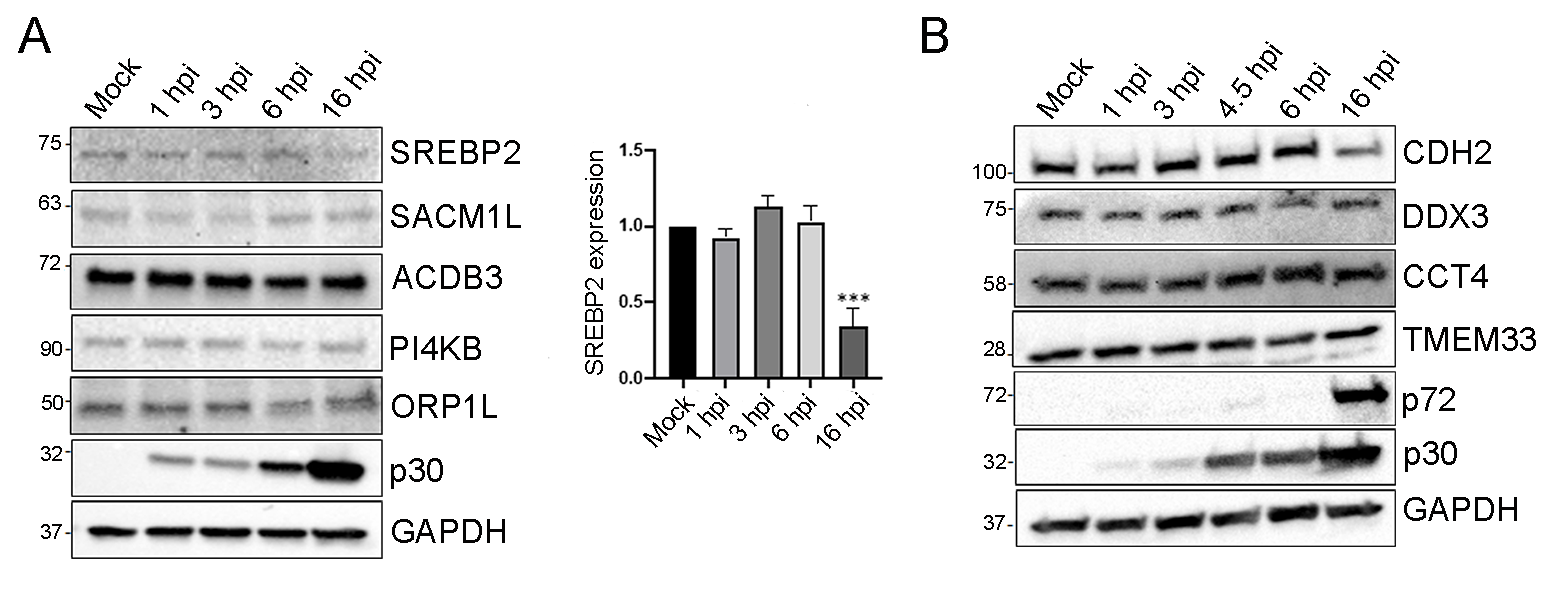

Supplement: Supplementary file 1 [file viruses-15-01098-s001.zip › Figure S4.tif]

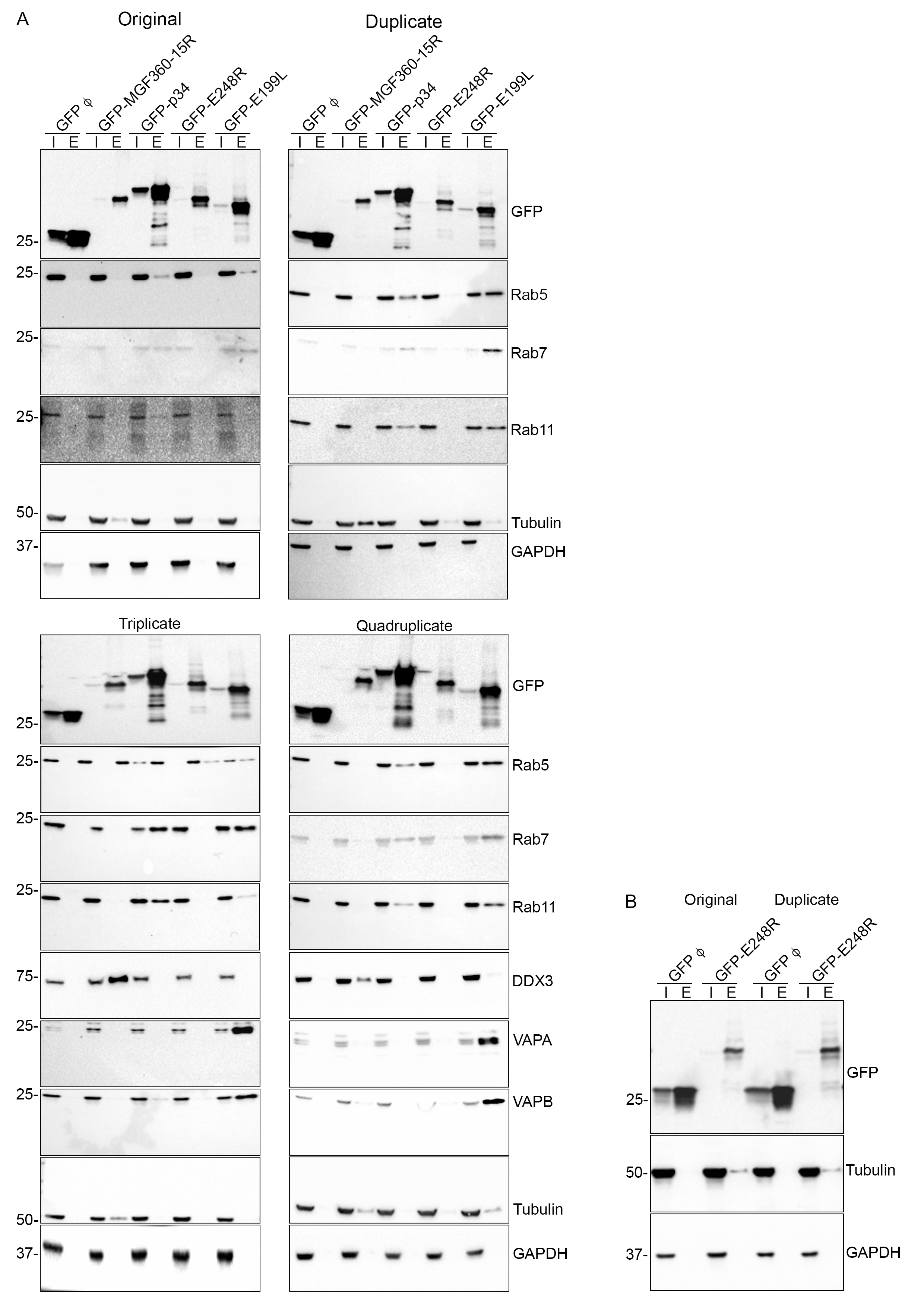

Supplement: Supplementary file 1 [file viruses-15-01098-s001.zip › Figure S5.tif]

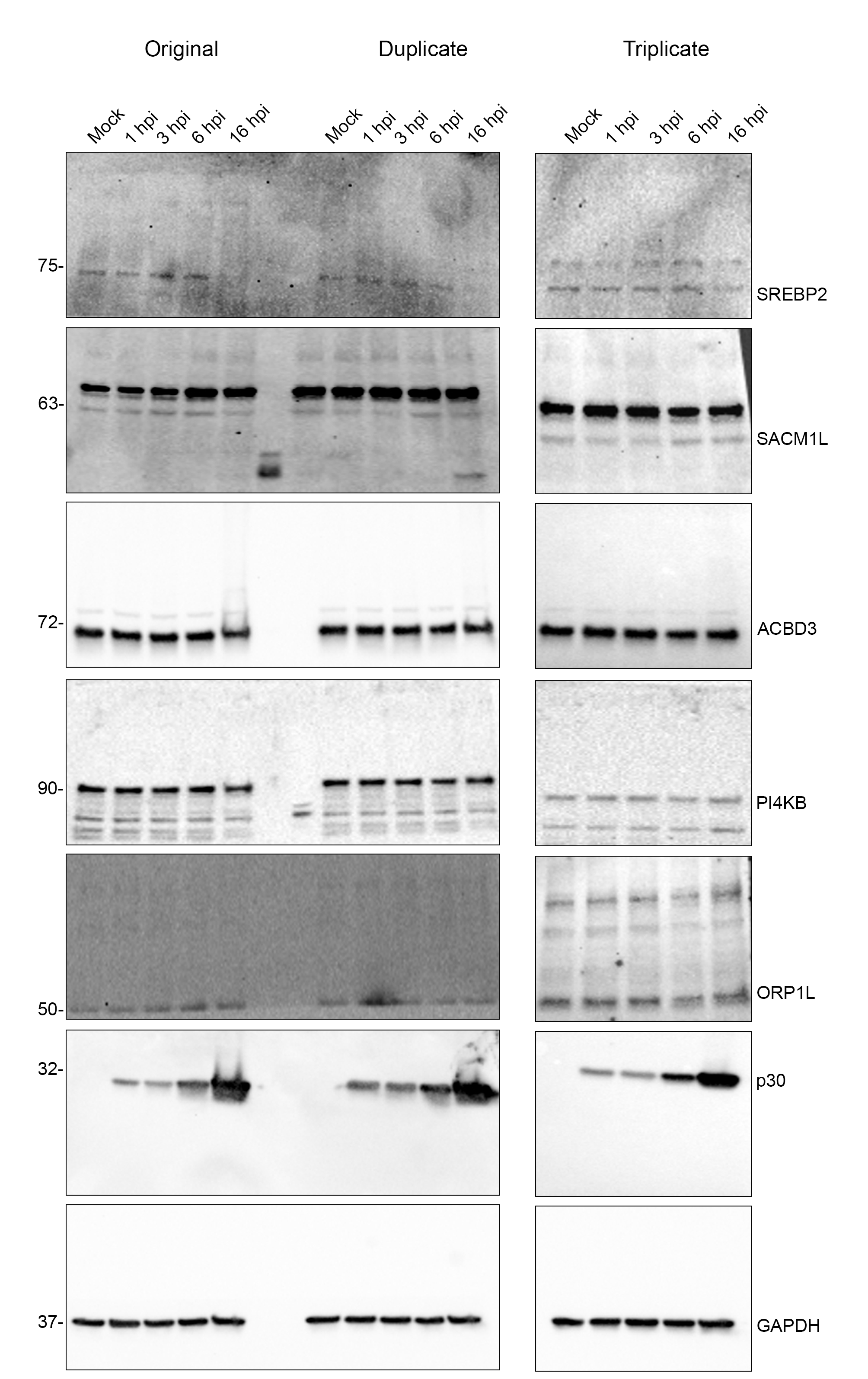

Supplement: Supplementary file 1 [file viruses-15-01098-s001.zip › Figure S6.tif]

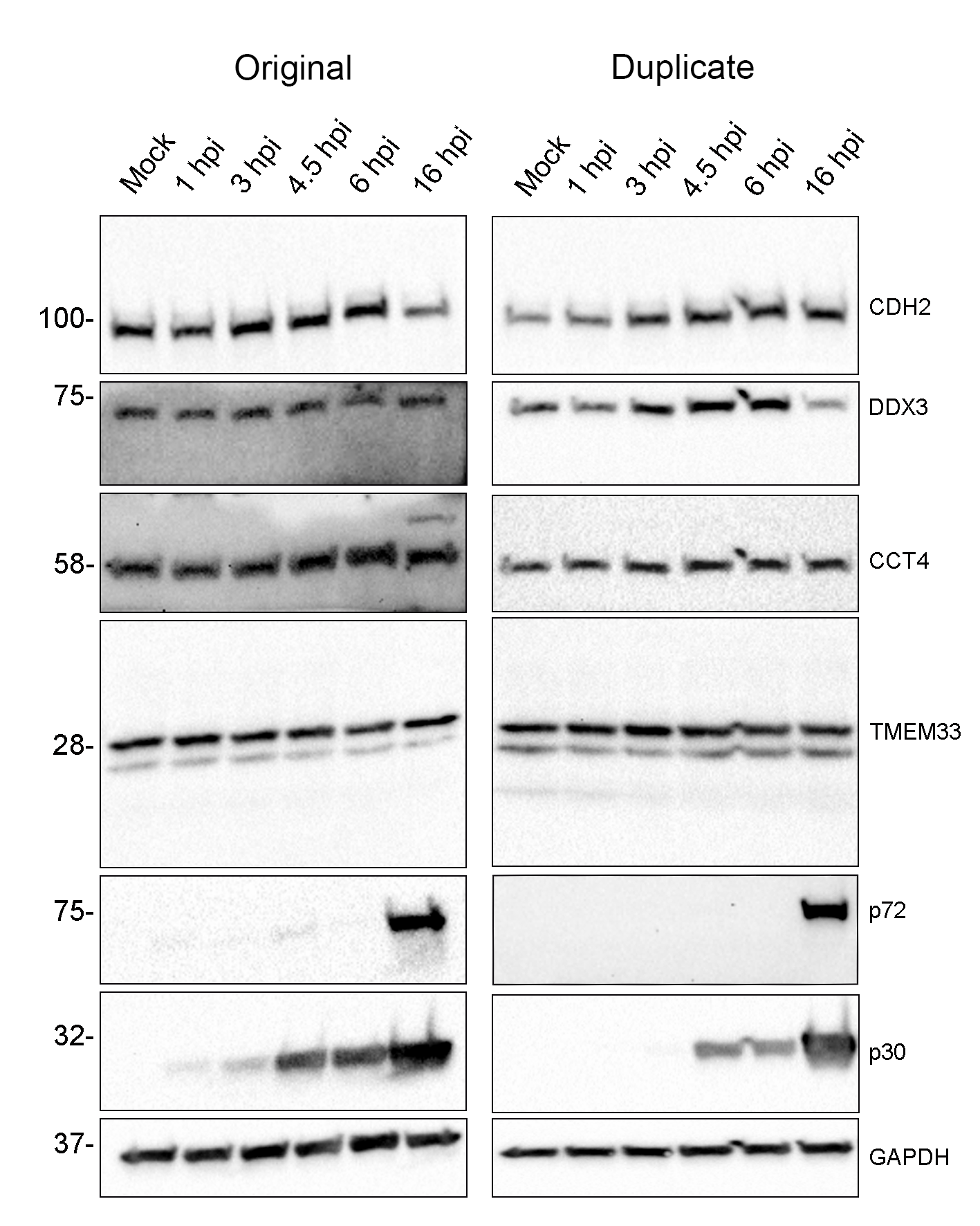

Supplement: Supplementary file 1 [file viruses-15-01098-s001.zip › Figure S7.tif]
